# Introductory Lecture to the Course 1841-2, Delivered in the Anatomical Theatre of the University of Pennsylvania, Thursday, November 4, 1841

**Published:** 1841-11-20

**Authors:** 


### BIBLIOGRAPHICAL NOTICE.

*Introductory Lecture to the Course* 1841–2, *delivered in the Anatomical Theatre of the University of Pennsylvania, Thursday, November* 4, 1841. By William E. Horner, M.D., Professor of Anatomy. (Published by the Class.)

We are happy to welcome the appearance of the first of the series of Introductory Lecture at the University of Pennsylvania for the current season. Such productions are too frequently intended for mere temporary effect, and instead of being, as they ever should be, an expose of the progress of the sciences of which the courses are designed to treat, a statement of the peculiar views or intentions of the lecturers in treating of those sciences, or a collection of appropriate remarks on the conduct and purposes of those who constitute the audience, and whose future career in life may be rendered more prosperous and happy by the lights of experience—they are too commonly designed to win an additional dollar, or to enhance an ephemeral reputation at the expense of the pecuniary or intellectual interests of the hearers, for the personal benefit of the speaker. In other words, an Introductory Lecture—like a discourse before a modern Lyceum—is, commonly, a thing of mere effect. Those which we have had the good fortune to hear and comment upon at the various institutions connected with the Medical School of Philadelphia, during the present year, have been, for the most part, exceptions to the general rule. That of Dr. Horner is peculiarly so. It is "a round unvarnished tale," principally designed as a defence of Special Anatomy—a branch in which this Professor stands unrivalled, at least on this side of the Atlantic.

Every prejudice of our heart is naturally enlisted in favour of our venerable Alma Mater, and our feelings—we frankly confess it—would lead us to view with favour any thing emanating from her bosom; but, having early adopted the motto "*Fiat justitia, ruat cœlum,*" we acknowledge neither friendships nor enmities in science, and shall not hesitate to be quite as captious in criticism upon Dr. H. as if he were a Professor of some distant school. It is with such feelings of perfect independence that we extract and comment upon certain passages of his address, dwelling, as in professional duty bound, rather upon its defects than its excellences.

We make these remarks because it has been whispered that the change in the editorial department of this Journal was likely to give it an anti-university character. It will intentionally oppose nothing but error and advocate nothing but truth, regardless of the accidental source of the one or the other. If it fail in its intention, its own folly will be its only apology.

Introductories are like the first lessons of the mother to the infant. The impressions which they make upon the youthful mind often endures for life; and they are therefore important and legitimate subjects of criticism; and in the execution of our duties as scientific critics, while our course in this, as on all other occasions, will be independent of all cliques and factions—all local interests—all cavil and querulousness—will prove any thing but non-committal.

Towards the commencement of the Lecture Dr. Horner remarks:

"General Anatomy is of comparatively modern origin, and has for its inventors, Haller, Bordeu and Bichat. It is especially under the influence of the latter that its traits have been more distinctly figured, and that it has assumed a first rate importance. To my talented and eloquent colleagues, the Professor of the Practice of Medicine, and the Professor of the Institutes, you will be indebted for a just appreciation of its value, and for the pointing out in extenso of its connexion with the laws of organic life, and their immediate dependence on it. General Anatomy is derived from the following facts: Every animal is an assemblage of many organs, each of which executes a particular function, and by a harmonious co-operation with others keeps the machine alive. A minute analysis of each of these organs proves that it is composed of many elementary textures, just as we find chemical substances made up by a combination of several gases, acids, alkalis and other matters. Moreover, it is ascertained that every organ of the body presents such textures in greater or less number, and in proportions exhibiting great varieties. In some organs certain textures only exist, in other organs other textures, but the individual textures, wherever placed, preserve their type of originality, and their character is therefore universally preserved, notwithstanding a varied association with other textures, according to the organ to be constructed.

"Thus constituted, each tissue or texture, and each organ, are possessed of their specific vital properties, in which they differ to a remarkable degree from other textures and from other organs, in the phenomena and in the intensity of their life. Cartilages, tendons, and bones have but very little life, but the muscles and the skin have it very copiously. Moreover, each tissue has its peculiar modification of vitality, on which repose secretion, exhalation, absorption, and nutrition. The blood is the common store-house from which each chooses what suits its nature, and rejects other matters."

We think that, in the passsge just quoted, Professor Horner shares the error common to Bordeu, Bichat, and most general anatomists, in regarding the complication of tissues observable in the more complex animals after their organization has advanced beyond its first elementary stages in the fœtus, as constituting more absolute and fundamental specific distinctions between one tissue and another, than are found to exist in nature. It is certainly difficult to prove that "every animal is an assemblage of many organs," nor do we believe this to be the fact. An organ, in anatomical language, if we rightly comprehend the meaning of the term, is a portion of the living apparatus designed for the more perfect performance of some portion of series of vital functions. Now, in the porifera, the simple meducæ and the hydra, as well as in the germs of all the superior animals during the first moments of their existence, it is impossible to demonstrate the existence of any special organ. These living beings are all apparently composed of one single organ, and that organ is a cell or a congeries of cells; in other words—*cellular tissue.* Yet animals of this simple texture perform a function which is equivalent to the sum of all the vital functions, not excluding those which by a forced construction, authors have arranged under the head of the "animal functions," but which are just as peculiarly vital as are the most simple offices of vegetative life. They are capable of conveying to the consciousness the impression of external objects, and they act under the control of the will. They imbibe and assimulate —they secrete and transpire; and these four processes—probably the same in generic character—embrace all that is generically essential in all the vegetative functions, *so called.*

It is perhaps unfair to make peculiar views the basis of critical remarks on others, but these observations are necessary as exponents of our intended commentary of the proper mode in which the all-important and fundamental study of anatomy should be introduced to the student, and we must enter a protest against the comparison between the complexity of tissue observable in complex organs, and the combination of ingredients in a chemical admixture. In the former case, as Dr. H. pertinently remarks, all the tissues composing the organ, "wherever placed, preserve their type of originality." In the latter, all originality is lost. The tissue, if it deserves the name of a *species* at all, (which we seriously doubt,) preserves its specific character every where ; while, on the contrary, the chemical ingredient, when in combination, loses all signs of its identity.

The whole tendency of modern transcendental anatomy urges us to the conclusion that the origin of all animated beings is an organized cell, containing an organized fluid, which cell and fluid are capable of constructing, by means of their vital reaction, all the peculiarities of each species, tribe, or race. The whole course of modern surgical pathology tends equally to the conclusion that, at all stages of animal existence, any organ may be reduced in part or in totality, to the embryonic condition of a simple cell or congeries of cells—organized, and containing an organized fluid—in other words, to the condition of simple cellular tissue. It is equally plain, from the history of morbid changes in fractures, anchylosis, ossifications of the arteries, the brain, the liver, &c., that any complex organ, after being thus reduced, in part, to the condition of simple cellular tissue, *may* regain its original structure by the operation of the same laws that originally produced their construction under the operation of the vital energies of that simple tissue, which is the fundamental seat of life in all the organs. For these reasons, we look upon the several tissues of the human body not as species, but as mere *varieties* of structure.

These views, which may be regarded as original by most, are faintly shadowed forth in the opinions of Haller on the elementary fibre, and have received important support from the recent explorations with the microscope in the hands of those who have not neglected the multitudinous errors into which that extremely delicate and somewhat treacherous instrument is prone to lead the observer. Haller was a generalizer, and, although the most industrious application of a long life to the prosecution of science did not enable him to foreclose the labours of his successors in the field of Physiology, his suggestions have been the foundation of most of the researches of his suscessors to the present time. They have directed the course of many successful investigators who probably have never been familiar with his works.

Bichat, on the contrary, was a specialist, notwithstanding the generality of many of his anatomical conclusions; and he was the father of those doctrines which have elevated mere varieties of tissue into distinct species of structure, to the no small injury of our favourite branches of study, surgical therapeutics, and general physiology. In the present state of the prevalent systems of medical instruction, it is to the philosophical naturalist, rather than the physician, that we are to look for that improvement in the knowledge of the fundamental principles of organo-genesis (if we may be permitted to coin a technicality) upon which, alone, it is possible to construct a stable theory of pathological anatomy.

It is perhaps, due to Dr. Horner to apologise for making a mere phrase in an introductory lecture the text of so much comment. We will return to the subject upon some future, and, it may be, more suitable occasion.

The following remarks on oral lectures and ocular demonstration are peculiarly pertinent, but call for a word of comment.

"There is no department of knowledge, let it be ever so ably treated of in books, which may not be learned with additional facility, by oral instruction from a competent teacher. The interest which a public speaker excites, always produces a more lively and pleasant attention, than any printed communication from him. But if this hold good in the most abstruse inquiries, much more valuable is the plan where an object is to be demonstrated. This opinion is so true in Anatomy, that the solitary student, with the most approved descriptions and plates, and with a subject at his command, will find it one of the most discouraging and perplexing studies that can be undertaken. Besides his want of skill in the merely mechanical separation of parts, he finds in every dissection, such a resemblance in the colour and shape of organs of different natures, that his mind becomes bewildered, and he feels an insurmountable difficulty in making a clear and satisfactory progress. These impediments to solitary study are not exaggerated ; they are witnessed every year and universally confessed by such as have been exposed to them. Such being the case, there is no competent substitute for demonstrations from a teacher, and, especially, when his lessons are illustrated by the means for a full course of Anatomy."

While acknowledging, in their fullest extent, the importance of oral and ocular instruction in the peculiar branch which is cultivated by the lecturer, we cannot avoid a fear that the first sentence of the above extract contains too sweeping an assertion.

The study and the lecture room are separate fields of action. Each is, when rightly employed, the theatre of a distinct series of observations. The dissecting room is the place where the most accurate and thorough knowledge of special anatomy is to be obtained, but the elementary student requires a certain amount of education before he is prepared to enter the dissecting room, without an unnecessary expenditure of time and money in the attempt to acquire information. Oral lectures and ocular demonstrations are unquestionable prerequisites. These are the advantages obtainable by pursuing our researches in large Capitals, where subjects are at all times obtainable at moderate expense; and, but for these, the study of special anatomy in the private office of the country practitioner, or within the walls of a college distant from cities, would be just as successful as in the halls of a university. But the case is different with many other branches of medical knowledge, and even with some of an anatomical character. When the student has acquired considerable acquaintance with the details of special or descriptive human anatomy, and wishes to enable himself to render that acquaintance available in physiological reasoning—without which it is, practically, almost useless—he must prosecute the subject of general anatomy; and here, he must resort to libraries and the study. There is no cabinet in America fitted to illustrate a thorough course of lectures on this branch. There is no memory in America that could carry from the lecture room more than a mere smattering and parrot-like idea of its principles. These require for their mastery the quiet of a secluded room, and deliberate reflection over the unchanging types, for the want of which no hasty notes can compensate. The lecture room, in studies of this character, is but the sign post to direct the course of the student on the proper road. The subject is not *taught* there—it is merely *indicated.* The same remark is true in relation to pathological anatomy. Surgical anatomy constitutes an exception. Like the branch in which Dr. Horner is so highly and deservedly distinguished, it is, in great degree, a matter of illustration with proper oral comment—a matter of history rather than deduction. Extending our view to only one other department of the multiform elements of medicine, it will be perceived by all who have outlived the early ambition that looked to two square feet of parchment as the *summum bonum*—a folly which we blush to acknowledge was once as strong with us as with our successors—that the materia medica, properly so called, which is a branch of descriptive natural history, may be as thoroughly taught in the private office, by the aid of suitable specimens, as it can be in any lecture room. It could be better, because more impressively taught in connexion with pharmacy, by the aid of books, in a proper druggist's establishment, under the jurisdiction of a competent medical teacher, than in either of the before named situations. Not so, therapeutics! This science requires the aid of oral lectures for the inculcation of principles, and the clinical ward for the reduction of those principles to the test of experience.

We have now said quite enough to enforce what is, perhaps, of little importance, our individual dissent from the proposition of the lecturer, that "there is no department of knowledge, let it be ever so ably treated of in books, which may not be learned with additional facility by oral instruction from a competent teacher." Less, we could not have said conscientiously. The great folly of the age is lecturing—in place and out of place—lecturing without any regard to the distinction between the proper subjects for solitary reflection, biblical research, ocular demonstration, and oral teaching. We have listened *for an hour*, to a complete analysis of "the genius of Shakspeare"—*for an hour*, to "the commercial history of lesser Asia!"—*for an hour*, to "the character of English poetry!"—*for an hour*, to "the age of Pericles!" We have *remembered* the *thesis* of an Edinburgh graduate, "De omnibus rebus et quibusdem aliis," and the *forty-eight lessons* in which the celebrated Mr. Hamilton communicated *a thorough knowledge of the French language!*

The Lyceums and popular Institutes now take the lead in this *labour saving* mode of instruction, but the medical profession has been long acquainted with the process—and this remark cannot be applied with justice to any one College or University. It cannot be even charged as a vice, for it belongs to the era, and no existing combination of individuals could stem the current. One of the most distinguished scholars of the day has declared this to be "the age of expansion!" Truly, in some directions, it is the expansion of a bubble!

We have been led off by our feelings from the text of this article, but cannot fear any misapplication of the latter remarks to the able Professor of Anatomy, whose branch, above all others, befits the demonstrative lecture room, and who moves within that theatre with a degree of reputation and success which needs no praise from us. But it is time to proceed:—

"One of the most common deviations from that mode of teaching Anatomy which I have said enjoys my own preference, is the one called Surgical Anatomy, which has its origin in a conviction of the value of uniting the study of Surgery, in the same Lectures to the study of Anatomy. Nothing certainly can be more valuable than such an union when it is formed at a proper period, but in the early stages of one's progress, in a difficult pursuit, it ought not to be doubted, that the easiest way of familiarizing the mind to its objects, is to keep them clear of extraneous matters, and to present them in a state the least incumbered with other considerations. Elementary ideas are to be first instilled, their combinations are next to be formed, and lastly is to be studied that complicated intertexture of tissues and parts which the human machine so universally presents. Mr. Colles, of Dublin, a surgeon deservedly distinguished for his skill and for his valuable contributions to the science of medicine, is one of the most potent advocates for beginning with Surgical Anatomy, in our investigations of the structure of the human body. He gives in his work on Surgical Anatomy, the following account of the systematic plan as generally adopted. 'An attempt to explain the nature and structure of the animal machine, by dividing the several parts of which it is compased into distinct classes, and then giving a detached and unconnected description of each class, without ever considering them as component parts of one organized whole, is as preposterous and unavailing, as to attempt to explain the mechanism of a watch by taking it to pieces and giving a separate description of every wheel and spring, without afterwards attempting to show by what contrivance the one moves the other, or how each wheel contributes by its particular motion, to regulate the general movements of the whole.'

"I have selected the preceding passage because it comes from very excellent authority, and because it represents the sum and substance of the most plausible objections to the usual systematic teaching of Anatomy. This passage is, however, itself a fair subject of animadversion, because it exhibits defectively, I will not say disingenuously, the case which it claims to represent. The phrase 'giving a detached and unconnected description of each class, without ever considering them as component parts of one organized whole,' presents an exceedingly fallacious view of this question, and is indeed an assertion very badly sustained by the facts themselves. Thus, I ask, what anatomist demonstrates a muscle without including its origin, its insertion, and its contiguity to other muscles? With blood-vessels and nerves we always point out the positive relations to other parts, and also their destination. The position of the heart in the thorax, and its contiguity to other parts, are invariably leading and prominent traits in its description. The liver, besides an immense deal of intrinsic anatomy, is rich in the considerations arising from its position and relations. I might in this way pass from organ to organ, and vindicate in succession the usage of the systematic Anatomist in regard to each, but this pursuit may be very properly suspended upon the analogies already presented. The insulated dissections of the muscles of the extremities, and of the trunk, appear to me to be the ground work of all the preceding objections; to the systematic Anatomist, however, the muscles are the frame, the foundation along with the bones, upon which he erects his superstructure and finishes his task in all its details."

In this extract, as it may appear, Dr. Horner gives, perhaps, a severe construction of the meaning of Mr. Colles. We see nothing like acting disengenuously, in the passage quoted; it bears rather the stamp of prejudice from undue enthusiasm. It is true that, judging by the uncertain test of memory, (for the question is not sufficiently important to warrant the expenditure of time in making direct reference to authority in a mere ephemeral review,) Mr. Colles generally estimates the claims of his favourite department at more than their full value; but when the special anatomist undertakes to illustrate his subject by dwelling upon the relation of any organ with its neighbours longer than is necessary to fix the true location of the part, he trenches upon the domain of surgical or general anatomy upon the one hand, or on that of physiology and pathology upon the other. That he should frequently do so, is proper, and indeed necessary, in order to enliven a branch which calls for an almost dead effort of memory. Whenever he can enforce a point by producing an association in the mind of the pupil he should unquestionably do so. In the teaching of a subject intrinsically dry, the popularity of a lecturer depends mainly upon the tact with which he introduces such illustrations; but if he carries the custom a little too far, his course assumes a mixed character, losing all proper cohesion; and, although it may be brilliant, it can never be solidly useful. We have listened to lectures of this character in former years. Sometimes, however, the result is different, and the teacher deals with his own thoughts and those of others, much as the compiler deals with authorities when he depends upon his *scissors*; a sprinkling of conjunctions with a few unimportant phrases being alone required to give *unity* to the labours of thousands. Lecturers of this class are by no means rare. They spare themselves the labour of thought, are totally guiltless of originality, and, when most successful, it may be said of them that they spend a life in "stringing pearls with twine." Under all circumstances, it is impossible for the teacher of special anatomy to engross with his course the necessary instruction in surgical anatomy. The thorough treating of two such subjects concentaneously by one man, is out of the question. If, then, a single individual be charged with both duties, it is only by the virtual duplication of his course that he can properly fulfil them.

But special anatomy is a fundamental and purely elementary study, without a correct knowledge of which, no branch of the healing art can be honestly and availably practised. surgical anatomy, on the contrary, is mainly useful in one only department of that art. It is not a purely elementary, but, to a certain extent, a transcendental branch.

When Mr. Colles attempts, as he unquestionably does,—gravely or unintentionally,—to underrate the importance of special anatomy, he undertakes to build a temple without the necessary foundation; and, were he to prosecute his idea, would inevitably bring the superstructure to the ground.

On the other side of the question, were the special anatomist to endeavour to fulfil the duties of the surgical anatomist, without dividing his labour between two distinct and, in great degree, independent courses, he would certainly fail in his purpose. Both modes of teaching to which reference is made in the text, are necessary to render a system of medical instruction even tolerably perfect—but, of the two, that employed by the special anatomist is decidedly the more important; for it is impossible to make the student familiar with the relations of parts in different regions of the body, unless he be previously acquainted with the history of the particular organs, portions of which only come legitimately under the notice of the surgical Anatomist.

We extract, with great pleasure, the following condensed description of the origin and present condition of the Anatomical Museum, partly that the constant increase of the collection does honour to the industry and skill of Professor Horner; of whom, as well as of its distinguished founder, it will prove a lasting monument; and partly that it awards some portion of the praise due to the magnificent liberality and noble affection of a lady to whom science is deeply indebted, and of whom our city has just reason to be proud. Our greatest regret in relation to this museum arises from the want of such arrangements as would render it at all times accessible, not only to the students of the college, but to the profession and the scientific in general.

"Let us now pass from the consideration of the manner, to that of the sources and of the means from which your instruction is to be derived. One of the most prominent is the Anatomical Museum. Since the accession, in 1792, to the Chair of Anatomy by the celebrated Wistar, an unceasing object has been to improve and to enlarge this collection. At his death, in 1818, his amiable consort, bearing in mind the extreme interest which he had always felt for the advancement of his chair, and for augmenting the facilities of Anatomical instruction, became convinced that it was his intention to leave his cabinet to the University, and that the bequest would have been made had his sickness not passed through so rapid a course. Mrs. Wistar, therefore, determined to manifest her affection for the memory of her deceased husband, and the interest she felt in the scene of his labours, by purchasing at her own expense and presenting to the University his collection. This she did much to her own credit, and to the satisfaction of the friends of Dr. Wistar's reputation.

This collection consisted principally in a very fine series of dried preparations of the Sanguiferous and Lymphatic Systems, of Corrosions, and of Models in wood on a very magnified scale. The preparations of the Lymphatics are, for the most part, from the school of the celebrated Mascagni, having been imported from Italy about 1812. The other articles were made in Philadelphia. In the year 1834, the Managers of the Pennsylvania Hospital, with a commendable liberality, gave to the University their Anatomical Cabinet, consisting in preparations; in Models in wax, made by Dr. Chovet; and in Plaster Casts and Crayon drawings, both of which were originally sent as a present from Dr. Fothergill, of London, in 1762, being brought by Doctor William Shippen, the first Professor of Anatomy, but then a young man, and about settling in Philadelphia.

With an extensive foundation thus laid in the liberality of Mrs. Wistar, and of the Pennsylvania Hospital, it has been my unceasing care to keep those articles in a state of good preservation, and to add to their number. The extensive accommodations of the present Medical Hall, erected upon the ruins of two others, and the amplitude of the room allotted to the Anatomical Museum, having afforded a strong incentive and a great opportunity for exertion, a numerous series of wet preparations, forming now the body of the Museum, has been made, and includes a very large number of the most interesting specimens of morbid anatomy and of minute structure."

On the concluding page of the lecture, after some notice of distinguished alumni now holding professional chairs in other cities, we find the following happily expressed and manly allusion to other rival teachers within our own limits.

"Even within a few minutes walk of where we are now assembled, there are gentlemen of justly merited and distinguished reputation, our friends and our former pupils, who, emulous of the renown attached to the institution from which an humble individual is now addressing you, have their aspirations excited to the same career of honour and of usefulness. Though on the field of competition, they are no doubt justly and liberally so, and remote may be the period when any sentiment or language may emanate from either side, which may offend the feeling of professional sensibility—set a pernicious example to younger members—or detract from the high dignity which medicine has attained in this city. Neither school, I believe, is prepared to do, or rather to attempt to accomplish, by vilification and reproach, what it despairs of accomplishing by hard work."

We will close this unusually protracted article with the remark that one of the most obvious defects of the system of medical teaching, in most of the schools, is the want of a primary course of lectures on the nature and relations of the different medical sciences, and on the classes of organs constituting the human machine which forms the subject of those sciences;—a course which should be clothed in popular, instead of technical language, in order that it may be intelligible to the mere beginner. From such a course, the pupil would pass to the halls of our colleges with a power of comprehension usually acquired at the sacrifice of the chief advantages of the studies of the first winter. It is almost painful to witness, *as it was once to feel*, the effort of the tyro in endeavouring to follow the lecturer when informed that *this process* gives origin to the coraco-brachialis muscle, many weeks before he has formed his first idea of the nature of a muscle, or when told that *that foramen* gives passage to the "portio dura," months before he is taught properly to distinguish between a nerve and a tendon. But when we find him gravely listening to disquisitions on the nature and treatment of fever or inflammation before he has pushed his anatomical studies beyond the bones of the head, his chemical researches further than the laws of simple affinity, as displayed in binary compounds, and his knowledge of the materia medica as far the "*the middle of emetics*," the pain is tempered by the sense of the ludicrous, and it becomes dangerous in a grave assembly even to glance at the puzzled countenance of the intelligent and ambitious "first course student," striving to translate the unintelligible. Our system has alarming faults at both extremities. It neither commences systematically nor proceeds to the extent which the wants of elementary instruction demand. No man feels this truth more strongly, or acknowledges it more frankly than the learned and laborious Professor upon whose Introductory Lecture so many remarks have been *suspended*. But, by those who hold the power, these faults are considered as inseparably dependent on the unwillingness of the class to endure a longer period of study. In this we believe, and always have believed them to be mistaken; but—granting the truth of the position,—whence is that unwillingness derived?—assuredly from the neglect of the teachers in instructing the class as to their true interests. A proper course of lectures, such as we have named, would revolutionize any class in the country within three years. The times are ripe for advancement; and the school that would now assume or preserve its pre-eminence, must advance with the age. Some tendency to this may be perceived in the movements of the last six years. It may be difficult to induce men to sacrifice some little immediate interest and labour for what may be regarded as doubtful in result, but if those whose advantages are already strongly concentrated, and in whom it would be desirable for the profession *that still further advantages should be concentrated*, refuse to run the risk of the experiment, there are others always at hand who will. If one city declines the test, another will assume that responsibility;—if one college refuses it, another will undertake it;—and if no one now existing will make the move, others will be created for the purpose. Even if all should be induced "to follow in the footsteps of their predecessors," then individuals will take the helm, and in a few years the whole system of collegiate medical iustruction will totter to its foundation.

We are no friends to *medical agrarianism*, however deeply attached to well-ordered *republicanism* in science; and should deeply deplore the further diffusion of the right to confer degrees—already far too widely extended—unless, by the action of the intelligence of the whole profession, a proper line of demarcation can be drawn between the diploma that *is* a certificate, and the diploma that merely *professes to be so*. It is surprising that these truths, rendered so plain by the events of the last few years, should still escape the attention of medical politicians; but the vision of the politician is too often bounded to the range of the next election day. But we have wandered far from the text of our discourse, into questions which will call for attention in another place. One word and we have done. The proper preface to the study of special anatomy is a condensed view of general anatomy and physiology, clothed in proper language. By such a view, a study usually as dry to the beginner as the definitions of signs at the commencement of a treatise on algebra on the old fashioned English model, may be rendered a delightful course of observation, in which, from the very commencement of osteology, every fact will be enlivened and impressed by many natural associations, without the necessity of "*beginning*" the study in the manner recommended by Mr. Colles, and very justly censured by Dr. Horner.

